# Genomic prediction of wild-derived powdery mildew resistance for strawberry (*Fragaria × ananassa*) pre-breeding

**DOI:** 10.1038/s41437-026-00857-2

**Published:** 2026-06-17

**Authors:** Attiq ur Rehman, Hanni Kärkkäinen, Alan H. Schulman, Napoleon Vargas Jurado, Ismo Strandén, Tuuli Haikonen

**Affiliations:** 1 Natural Resources Institute Finland (Luke), Helsinki, Finland; 2 Doctoral Program in Plant Sciences, University of Helsinki, Helsinki, Finland; 3 Luke Doctoral Program, Natural Resources Institute Finland (Luke), Helsinki, Finland; 4 Boreal Plant Breeding Ltd., Jokioinen, Finland; 5 Viikki Plant Science Centre, University of Helsinki, Helsinki, Finland; 6 Institute of Biotechnology, University of Helsinki, Helsinki, Finland

**Keywords:** Plant genetics, Plant breeding

## Abstract

Genomic prediction (GP) has become an essential tool for accelerating modern plant breeding, particularly for complex traits. We evaluated different GP approaches for pre-breeding in thirteen biparental strawberry families derived from crosses between *Fragaria virginiana* and *Fragaria chiloensis*, focusing on resistance to powdery mildew (PM) in both leaves and fruits. Five-fold cross-validation using Genomic Best Linear Unbiased Prediction (GBLUP) for combined-year data yielded mean predictive abilities (PAs) of 0.56 and 0.36 for leaf and fruit resistance, respectively. Family-based GBLUP analyses showed higher PAs for closely related families, ranging from 0.10 to 0.89. Simulations identified key parameters: training sets comprising 40% of the population provided stable predictions for both traits, while ≈8300 SNPs were sufficient for predicting leaf resistance. However, PA for fruit resistance remained consistently low regardless of marker density. We then compared GBLUP with a marker-assisted model that iteratively incorporated the major resistance loci as fixed effects. This strategy increased PA by ≈10–30% for leaf resistance and ≈25–47% for fruit resistance across models. We further applied cross-environment forward prediction in independent greenhouse and separate field validation trials. The GBLUP model maintained substantial PA when trained on either the full population or a 40% subset, demonstrating robustness in predicting new genotypes across distinct environments. Our findings highlight the importance of taking trait genetic architecture into account to enhance PAs for PM resistance in strawberry. Together, these results provide evidence-based thresholds for training population size and marker density, offering a framework for efficient implementation of genomic selection in strawberry pre-breeding populations.

## Introduction

Powdery mildew (PM), caused by the obligate biotrophic fungus *Podosphaera aphanis*, is a major fungal disease affecting garden strawberry (*Fragaria × ananassa*) production worldwide. In addition to garden strawberry, a range of susceptibility to *P. aphanis* among different accessions of other *Fragaria* species has been reported, including *F. vesca* (Iwasaki et al. 2021), *F. virginiana* (Kennedy et al. 2013), *F. moschata* (Shi et al. 2023), and *F. iinumae* (Shengrui et al. 2012). In the cultivated strawberry, PM disease leads to unmarketable strawberry fruit and significant yield losses if left unmanaged (Peries 1962). The pathogen causes disease on different plant organs, including leaves, stolons, flowers, and fruits, reducing both the quality and quantity of the harvest (Paulus 1990; Maas 1998). Common leaf symptoms include upward curling of leaflet edges, the appearance of powdery white mycelia, and in severe cases, necrotic lesions on leaflet margins. Infection of flowers and fruits results in poor fruit quality and arrested ripening (Nelson 1996; Amsalem et al. 2006).

PM poses a serious challenge for both open-field and tunnel growers worldwide, including in the Nordic countries, where disease outbreaks before harvest are frequent (Davik and Honne 2005; Kennedy et al. 2013; Aldrighetti and Pertot 2023). It is also problematic for growers of everbearing cultivars, due to their extended harvest period (Carisse and Bouchard 2010). Despite the reliance on foliar fungicides to manage PM, their efficacy has been undermined by the emergence of fungicide-resistant pathogen populations (Palmer and Holmes 2021). Moreover, the increasing demand for reduced agrochemical use in agriculture necessitates the development of alternative sustainable strategies, including breeding for genetic resistance.

The garden strawberry is an allo-octoploid (2n = 8x = 56) species that originated from hybridization between *F. chiloensis* and *F. virginiana* approximately 300 years ago. Wild populations of these progenitor species harbor valuable genetic diversity for horticultural traits, including resistance to pests and diseases (Hancock et al. 1991; Cameron et al. 1993; Kennedy et al. 2013). Previous breeding efforts have identified these wild resources as potential sources of resistance to PM. For example, *F. virginiana* subspecies *virginiana* has shown notable resistance, whereas *F. chiloensis* exhibits a range of susceptibilities (Hancock et al. 2001; Kennedy et al. 2013). Previously, we reported a major Quantitative Trait Locus (QTL) on chromosome 3B associated with stable PM resistance in a pre-breeding population derived from these wild materials (Rehman et al. 2025).

In the germplasms of cultivated strawberry, PM resistance is a quantitative trait with moderate to high heritability, influenced by multiple small-effect loci and environmental interactions (Simpson 1987; Davik and Honne 2005). The genetic basis of resistance available in the cultivated gene pool remains less understood, particularly for fruit PM resistance, where no stable QTLs have been identified (Cockerton et al. 2018; Sargent et al. 2019; Lynn et al. 2024). Most studies suggest that leaf PM resistance is governed predominantly by additive genetic effects, although non-additive effects have also been observed (Daubeny 1961; Hsu et al. 1969). This complexity has made it difficult to develop robust resistance through traditional breeding methods, which often rely on phenotypic selection or marker-assisted selection (MAS). While MAS can be effective for traits controlled by a few major QTLs, its utility is limited for complex traits like PM resistance, where numerous loci with small effects contribute to the total phenotypic variation. The challenge of navigating polygenic architecture is common to many outcrossing, heterozygous species, making the development of predictive frameworks a critical part of modern crop improvement.

In recent years, genomic selection (GS) has emerged as a powerful tool for improving quantitative traits in crop plants (Alemu et al. 2024). Unlike conventional gene mapping approaches, which identify specific loci associated with a trait, GS uses genome-wide marker data to predict Genomic Estimated Breeding Values (GEBVs) (Nakaya and Isobe 2012). This approach is particularly valuable for traits governed by numerous loci with small effects, as it captures the combined influence of all markers, regardless of their individual significance. For complex traits like PM resistance in strawberry, whose reliable assessment requires phenotyping in a labor-intensive timeseries and whose expression is influenced by environmental conditions, GS offers an excellent opportunity to accelerate breeding cycles and improve selection accuracy. Previous studies have demonstrated the feasibility of GS for PM resistance as well as for many other traits in garden strawberry, including other disease resistances, fruit quality, and yield (Gezan et al. 2017; Pincot et al. 2020; Osorio et al. 2021; Yamamoto et al. 2021; Tapia et al. 2022; Alam et al. 2024; Sleper et al. 2025). However, as previous studies have largely focused on elite or cultivar materials, the potential of GS to facilitate the integration of polygenic traits from wild-derived pre-breeding germplasm remains largely unexplored. Such studies are needed to facilitate integration of wild polygenic traits into strawberry breeding programs and, in general, could provide templates for utilizing wild diversity in other polyploid crops, too.

Our previous study has found that strawberry PM resistance available from the wild progenitor species via species reconstruction is genetically controlled with at least one major QTL associated with resistance to PM in both leaf and fruit tissues, though several other QTLs are also involved in conferring high levels of resistance (Rehman et al. 2025). The possible selection strategies of these major and minor QTLs for introgression breeding remain unexplored. In this study, we inspected genomic prediction (GP) as a tool for better assessment of wild-derived resistances in strawberry. We predicted PM resistance across a structured multi-family pre-breeding population, referred to as the reconstructed strawberry (ReC) population, and hypothesized that genetic relatedness between families would fundamentally enhance predictive ability for disease resistance in both leaf and fruit tissues.

We proposed that systematic optimization of training parameters would reveal distinct GP requirements for these traits. We further hypothesized that integrating significant markers from Genome-Wide Association Study (GWAS) into GP models would improve breeding value predictions in the population. Lastly, we assessed the robustness of the GP models by validating predictions in small subsets of the ReC population across two independent environments. This work represents a critical step toward implementing GS for directing the use of pre-breeding materials in breeding for more resistant garden strawberry cultivars, offering possible workflows that can be adapted to improve other complex traits in many plant species and breeding systems.

## Materials and methods

### Plant material, field trial, and datasets

The ReC population consisting of 13 biparental families (PPPS_1 to PPPS_13) developed through interspecific crosses between accessions of the progenitor species *F. virginiana* and *F. chiloensis* was evaluated in a field experiment at the Horticultural Research Station of Natural Resources Institute Finland (Luke) in Piikkiö, Kaarina, southwestern Finland (60°23′ N, 22°33′ E), as detailed in our previous work (Rehman et al. 2024). The ReC seedling individuals were propagated into plots of four plants repeated in two blocks. Additionally, plots of five control cultivars were repeated in each row to allow statistical control of spatial variation. The final phenotypic and genotypic datasets for 298 individuals of the ReC population come from our earlier GWAS and includes biweekly timeseries of leaf powdery mildew severity (leaf PM) evaluations for three seasons (2020, 2021, 2022) and seasonal (1–2 observations per season) for fruit powdery mildew severity (fruit PM) for two seasons (2021, 2022) (Rehman et al. 2025). For the year 2022, only one block was observed for both traits. For fruit PM, Best Linear Unbiased Estimators (BLUEs) were computed both year-wise and by combining the 2 years of data. For leaf PM, pre-calculated BLUEs for four aggregates (Endpoint, Mean, Area Under Disease Progress Curve (AUDPC), and Area Under Disease Progress Stairs (AUDPS)) were considered (Dataset, 10.5281/zenodo.20505163). A publicly available ‘IdeTo’ spreadsheet was used to compute the AUDPC and AUDPS aggregates according to Eqs. (1) and (2), respectively (Simko and Piepho 2012; Simko 2021).

1 $${\rm{AUDPC}}=\mathop{\sum }\limits_{i=1}^{n-1}\,\left(\frac{{y}_{i}+{y}_{i+1}}{2}\right)\times \left({t}_{i+1}-{t}_{i}\right),$$

where ${y}_{i}$ is the disease severity at the i-th time point, ${t}_{i}$ is the time at the i-th observation, n is the total number of observations.

2 $${\rm{AUDPS}}={\rm{AUDPC}}+\left(\frac{{y}_{1}+{y}_{n}}{2}\times \frac{{t}_{n}-{t}_{1}}{n-1}\right)$$

where the terms ${y}_{1}$ and ${{y}}_{n}$ are the disease severity scores at ${t}_{1}$ and ${t}_{n}$ that refer to the initial and final week numbers of the time when the disease scores were taken, respectively. The n represents the total number of observations.

### Narrow-sense heritability estimation

For each ReC family, the realized additive genomic (**G**) relationship matrix (VanRaden 2008) was computed via the *'A.mat()'* function through the ‘sommer’ R-package (Endelman 2011). This computation used a subset of genotypic data containing all markers, but only for the individuals belonging to that specific family. Using these family-specific additive matrices and the corresponding aggregated phenotypic data, a linear mixed model was fitted independently for each family using the ‘lme4breeding’ R-package (Covarrubias-Pazaran 2024). In each model, the genetic variance among individuals was captured by modeling individuals as a random effect with a covariance structure defined by the respective **G** matrix. Environmental variation was accounted for by including block, row, column, and their interactions with individuals as additional random effects, while year was treated as a fixed effect. After fitting the model for each family, variance components were extracted, and the narrow-sense heritability (*h*^2^) for each family was calculated using the formula (3):

3 $${h}^{2}=\frac{{\sigma }_{A}^{2}}{{\sigma }_{A}^{2}+\,{\sigma }_{E}^{2}\,}$$

where ${\sigma }_{A}^{2}$ is the additive genetic variance multiplied by the average diagonal d_A_ of the **G** matrix, and ${\sigma }_{E}^{2}$ is the environmental variance. This process was repeated independently across all families, resulting in family-specific heritability estimates. In addition, the number of individuals per family was recorded and reported alongside the heritability estimates to reflect the genetic sample size contributing to each calculation.

### Genomic prediction

The GP analysis was performed using the ‘rrBLUP’ package (Endelman 2011) in the R environment (R Core Team 2020). The analysis followed the mixed model formula (4) of GBLUP implemented in the *'kin.blup'* function:

4 $${\bf{y}}={\bf{Xb}}+{\bf{Zu}}+{\bf{e}}$$

where ${\bf{y}}$ represents the vector of adjusted BLUEs for respective leaf PM and fruit PM, ${\bf{X}}$ is a full-rank design matrix for the fixed effects ${\bf{b}}$, ${\bf{Z}}$ is the design matrix for random effects **u**. The random effects were assumed to follow ${\bf{u}} \sim N({\bf{0}},{\bf{K}}{\sigma }_{u}^{2}$), where **K** is the genomic relationship matrix, and ${\sigma }_{u}^{2}$ is the additive genetic variance. The residuals were assumed to follow a normal distribution with mean zero and equal variance ${\sigma }_{e}^{2}$, i.e., ${\bf{e}}\sim N({\bf{0}},{\bf{I}}{\sigma }_{e}^{2})$. The genetic and residual variances were estimated using REML by the *'kin.blup'* function.

To assess the contribution of trait-associated loci to GP, we tested Marker-Assisted GBLUP (MABLUP) models by including selected SNPs as fixed effects. This was implemented using the *'mixed.solve'* function of ‘rrBLUP’ and the following mixed model Eq. (5):

5 $${\bf{y}}={\bf{Xb}}+{\bf{Wm}}+{\bf{Zu}}+{\bf{e}}$$

where ${\bf{y}}$ is the vector of adjusted BLUEs, ${\bf{Xb}}$ represents non-marker fixed effects, ${\bf{Wm}}$ represents fixed effects for the selected SNP markers, **Zu** corresponds to the additive genomic effects, and **e** is the vector of residuals. We assumed that ${\bf{u}}\sim N({\bf{0}},{\bf{K}}{\sigma }_{u}^{2})$ with the known genomic relationship matrix ${\bf{K}}$, and ${\bf{e}}\sim N({\bf{0}},{\bf{I}}{\sigma }_{e}^{2})$. The matrix ${\bf{W}}$ contains the genotype scores (−1, 0, 1) for the selected SNPs of the training individuals. The selected SNPs were excluded from the calculation of the **K** matrix to avoid double-counting their effects. Thus, the MABLUP framework was consistent across analyses, and differences among models were solely from the set of SNPs included as fixed effects. Two SNP selection strategies were used for inclusion as fixed effects in the MABLUP models, analogous to the “historic” and “de novo” approaches described earlier (Spindel et al. 2016).

Historic SNPs included: (1) top SNP having the highest *−*log(*p*) value, and (2) focal SNP, explaining the highest phenotypic variance explained (PVE%) across different years and across different disease aggregates and with better allelic balance, both sourced from the major QTL(s) reported earlier (Rehman et al. 2025). These SNPs were discovered using the full ReC population. The de novo SNPs were obtained from GWAS conducted exclusively within each training population during 5-fold cross-validation. Because historic SNPs do not preserve cross-validation independence, a second GWAS procedure was implemented using the Mixed Linear Model (Yu et al. 2006), in the *'GWAS'* function of the ‘rrBLUP’ package (Endelman 2011). In this approach, SNP discovery was repeated separately for each of the five folds using only the training portion of the population to ensure a rigorous and unbiased validation. The two most significant SNPs from each fold, based on their −log(*p*), were then selected and incorporated as fixed effects in the corresponding fold’s MABLUP model. The mean predictive ability (PA) of these models was computed as the correlation coefficient (*R*) between GEBVs and the estimated phenotypic BLUEs of the test folds for the models with top-most significant SNP (SNP1; highest −log(*p*) value) and the second best one (SNP2; second highest −log(*p*) value) as fixed effects.

### Cross-validation

We implemented the standard five-fold cross-validation protocol (Schrauf et al. 2021) for evaluating both GBLUP and MABLUP models with historic SNPs and repeated the cross-validation process 100 times for each model. Model performance was subsequently assessed by calculating the correlation coefficient (*R*) between GEBVs and the estimated phenotypic BLUEs across all validation subsets within each validation run.

### Random sampling procedures

To gain further insights on the influence of population size and marker density on GBLUP models, two sub-setting procedures based on random sampling without replacement were run to test the PAs by: (1) progressively decreasing training population size from the complete population to 10% of its individuals, and (2) sequentially reducing marker density from the complete marker set of 20,779 SNPs (Dataset, 10.5281/zenodo.20505163) to only 10% of the markers. These sampling procedures were repeated 1000 times using the functions *'sample()'* and *'set.seed()'* with a 3-digit seed in base R (R Core Team 2020). These analyses exclusively utilized GBLUP to establish baseline performance and to examine the optimization of each training design parameter. Model’s PA was assessed using the correlation coefficient (*R*) between the phenotypic BLUEs and the mean GEBVs, obtained via five-fold cross-validation. Visualization was performed using *ggplot* R-package (Wickham 2016).

### F-statistics and sib family prediction

Each family of the ReC population includes, on average, 25 full-sib individuals with interconnected pedigrees to other families. For this study, the F-statistics (F_ST_) for the degree of interconnection between families were calculated using *'genet.dis'* function in ‘hierfstat’ R-package (Weir and Cockerham 1984; Goudet 2005) (Dataset, 10.5281/zenodo.20505163).

The ability of the GBLUP model to predict related progenies was then compared by sub-setting the ReC population using the known pedigree information (13 families), and family-wise forward predictions, where one family was predicted at a time while all the remaining families were used for training. Both GBLUP and MABLUP models were compared for their abilities to predict sib-families.

### Cross-environment forward predictions using GBLUP

To evaluate the PA of our GBLUP model across environments, the ReC population (*n* = 298) was used as the training dataset to predict the performance of subsets of ReC genotypes in two independent validation trials: a greenhouse validation trial (GH trial; reported in Rehman et al. 2025, *n* = 77) and a new field validation trial (*n* = 80). The field validation trial was conducted at a neighboring field plot to the ReC experiment, but on a different soil type and in different years (2023–2024). Both the GH and the field validation trial followed completely randomized block designs and included three replicates of each ReC individual, as well as one and five control cultivars, respectively. A linear mixed model was fitted, and BLUEs were calculated for the mean Leaf PM scores for both the GH and field validation trials, and for Fruit PM scores for the field trial only, using ‘lme4’ R-package (Bates et al. 2015) (Dataset, 10.5281/zenodo.20505163). To characterize the relationship between environments, the phenotypic correlation between the initial field trial and the two independent trials was calculated from common genotypes. Predictions for individuals in each target environment were then generated using the previously described GBLUP model, and model PA was assessed using the correlation between observed and predicted values. To ensure an independent cross-environment forward prediction, any individuals present in the validation sets were removed from the ReC training pool prior to model fitting, confirming mutually exclusive partitioning of the training sets from the testing sets.

To further evaluate model performance in a realistic breeding scenario, an additional cross-environment validation analysis with a reduced training set size was implemented. From the sub-setting analyses described in this work, 40% of the ReC individuals were identified as the minimum effective training population size for reliable GBLUP predictions (see results). Based on this threshold, 1000 random subsets of 40% of the ReC population were generated from the ReC trial as training sets, and predictions were performed for the GH and validation trial. This cross-environment approach was used to assess the robustness of GP models for predicting new individuals for PM resistance across distinct experimental conditions.

## Results

### Combined year analysis revealed the highest predictive abilities for the GBLUP model among traits and years

**Fig. 1:**
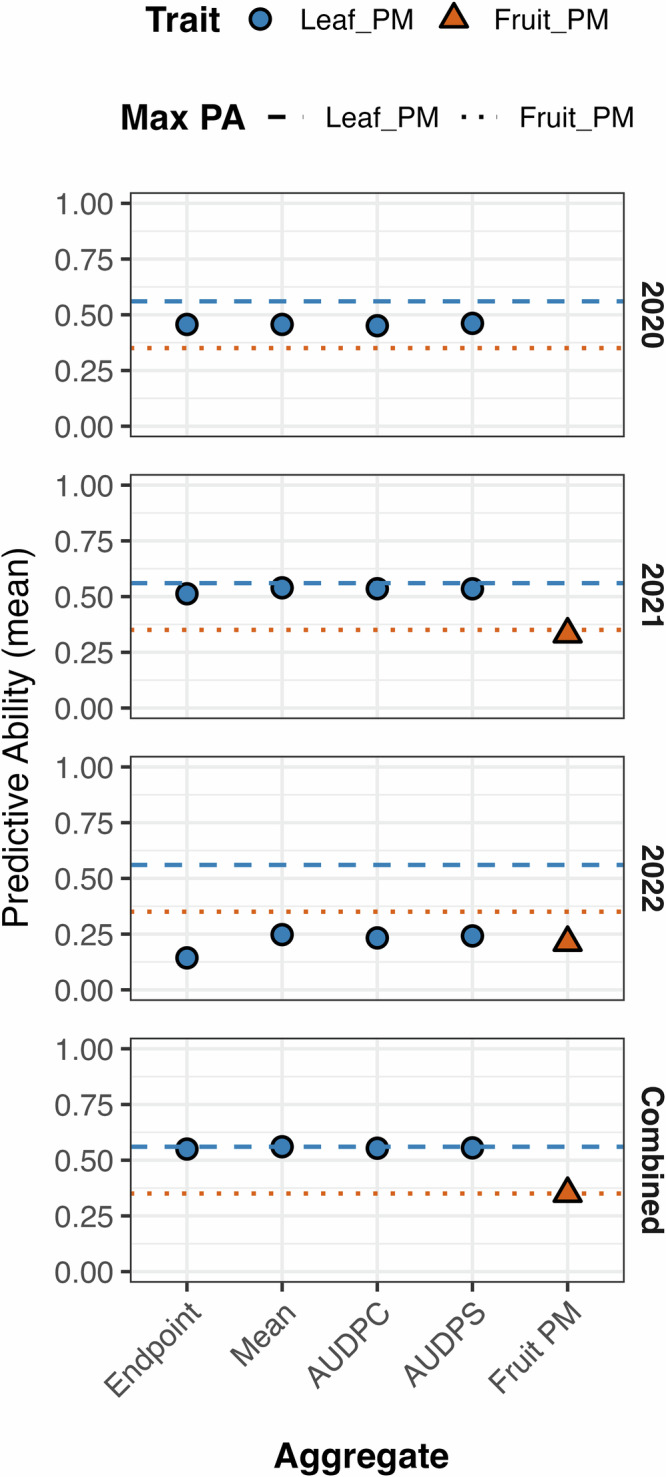
Model performance across different PM aggregates. Mean predictive ability (PA) for the Genomic Best Linear Unbiased Prediction (GBLUP) model after five-fold cross-validation with different leaf powdery mildew (PM) resistance aggregates (circles) and fruit PM resistance (triangles). Dotted horizontal lines indicate the maximum PA achieved for the respective trait.

The mean PA from the five-fold cross-validation of the GBLUP model over 100 replications, applied to each leaf PM resistance aggregate across 3 years, ranged between 0.14 ± 0.02 (mean ± SD) and 0.56 ± 0.01 (Fig. 1). In the year-wise analysis, the year 2021 demonstrated the highest PA (0.51 ± 0.01–0.54 ± 0.01), followed by 2020 (0.45 ± 0.02–0.46 ± 0.02), and 2022 (0.14 ± 0.02–0.25 ± 0.01). The differences between the PA for combined-year leaf PM resistance aggregates were minimal, with a range of 0.55 to 0.56. As the mean leaf PM BLUEs exhibited the highest PA, we chose this aggregate for subsequent analyses. Similarly, for fruit PM resistance, the combined phenotype across 2 years was selected for further analysis, as it demonstrated the highest PA of 0.36 ± 0.02, followed by the years 2021 (0.33 ± 0.02) and 2022 (0.21 ± 0.01) (Fig. 1). Although the 2021 dataset produced PAs comparable to the combined analysis, integrating multiple years of field data provided most reliable predictions, highlighting the importance of replicated phenotypic datasets for GP.

### Practical thresholds of marker density and training set size

**Fig. 2:**
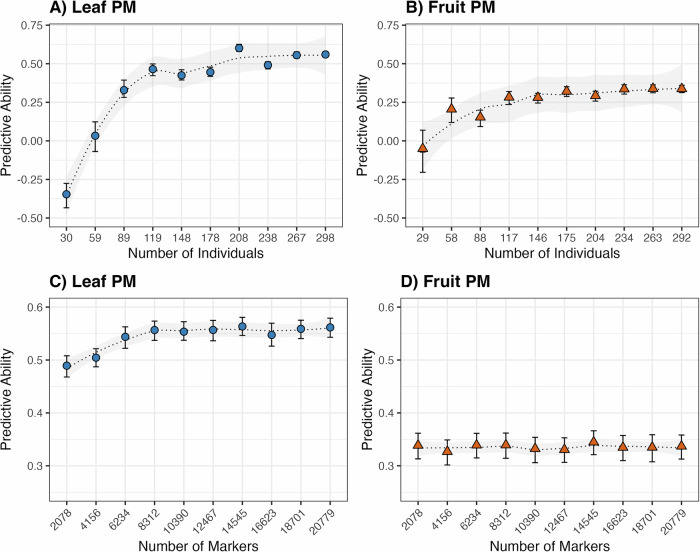
Influence of training set size and marker density on model performance. Effect of the number of individual plants (upper panel) and markers (lower panel) on the mean predictive ability (PA) of the Genomic Best Linear Unbiased Prediction (GBLUP) model tested for leaf mean powdery mildew (PM) (**A**, **C**) and fruit PM (**B**, **D**) resistance. Whiskers indicate standard deviations of the mean PA.

The impact of training population size and the number of markers on the PAs for both leaf (mean aggregate) and fruit PM resistances was inspected through a randomized sub-setting procedure. For testing the effect of training set size, the entire ReC population, comprising 298 genotyped individuals, was divided into 10 subsets, each progressively reduced by a random 10% of the population, until reaching a minimum of 30 individuals. The PA was found to be the lowest in the subset containing only 10% of the individuals, and a rising trend was observed as the training set size increased towards 100%. Any subset with more than 40% of individuals (*n* = 119) demonstrated a considerable PA of 0.40 or higher for mean leaf PM resistance (Fig. 2A). Similarly, for fruit PM resistance, the PA was lowest when only 10% of the individuals were used for prediction. For fruit PM, any training set with 40% (*n* = 117) or more achieved a PA of 0.27 or higher (Fig. 2B). Thus, for this data, for both resistances, 40% of the population, i.e., ≈120 individuals, was established as the minimum effective training set size for reliable predictions with GBLUP.

For analysis of the influence of SNP density, the final genotypic dataset, comprising 20,779 SNP markers, was sequentially reduced by removing 10% of the markers until reaching a minimum of 2078 markers (10%) from the full marker set. The PA for leaf PM resistance was the lowest with the sets of 2078 (10%) and 4156 (20%) markers. The PA increased until the marker set reached 8312 (40%) markers, where the PA was 0.55 for leaf PM resistance. Beyond this point, no notable improvement in PA was observed (Fig. 2C). However, no such trend was observed for the PA of fruit PM resistance when sequentially increasing the number of markers (Fig. 2D).

### **MABLUP outperforms standard GBLUP**

For both leaf and fruit PM resistance, PA from the baseline GBLUP model was compared with three MABLUP models, each incorporating significant QTL-associated SNPs iteratively. First, two historic SNPs were fitted as fixed effects from previously published work (Rehman et al. 2025). Subsequently, a de novo approach was followed, where SNP discovery was repeated within each training fold for respective traits, and the top two SNPs identified within each fold were fitted as fixed effects (Supplementary Figs. 1 and 2).

**Fig. 3:**
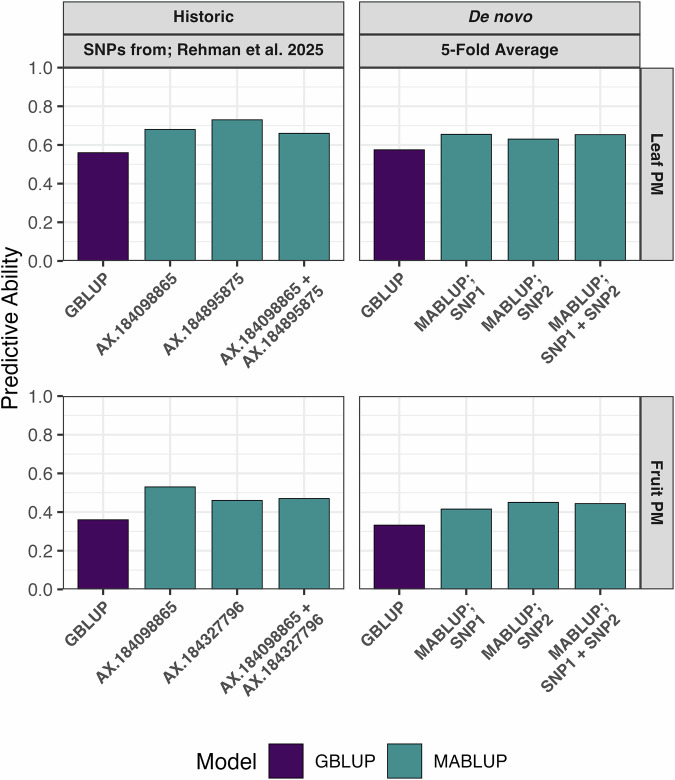
Predictive abilities (PAs) of the Genomic Best Linear Unbiased Prediction (GBLUP) and Marker-assisted Genomic Best Linear Unbiased Prediction (MABLUP) models for leaf PM (upper) and fruit PM resistance (lower). The left side of each panel shows the PAs achieved with the GBLUP model and when historic SNPs were fitted as fixed effects in the MABLUP model for the whole population, while the right side shows the mean PAs for GBLUP and the respective MABLUP model when de novo SNPs were fitted independently within each of the 5-folds of the training set.

The mean PA from the GBLUP model was 0.56 for leaf PM resistance. Notable improvements were observed when different SNP combinations were included as fixed effects. Incorporating the most significant leaf PM-associated historic SNP (AX.184098865), as identified by *p* value, from the major QTL on chromosome 3B (q.LPM.Rec-3B.2) increased the PA by 21.4% (Fig. 3). The second historic SNP (AX.184895875), selected based on its PVE%, instead of *p* value alone, yielded an even greater improvement of 30.4%. However, including both of these leaf PM resistance SNPs as fixed effects resulted in a 17.9% increase, which was lower than fitting either SNP individually (Fig. 3). The de novo strategy consistently re-identified SNPs from the same major genomic region on chromosome 3B across all five folds (Supplementary Fig. 1). Across folds, mean PA exceeded that of the GBLUP model and was comparable to the Historic MABLUP results (Fig. 3 and Supplementary Table 1), suggesting that the predictive signal from the chromosome 3B region is highly robust and reproducible across all independent training subsets. Like the Historic MABLUP models, including both SNPs in the de novo folds did not lead to notable gains, but the PA remained stable and stayed often close to the best MABLUP with only a single fixed SNP (Fig. 3).

The mean PA from the GBLUP model was 0.36 for fruit PM resistance. We observed a gain in PA with the MABLUP model by individually incorporating two historic SNPs (AX.184098865 and AX.184327796, both from the QTL q.FPM.Rec-3B on chromosome 3B) as fixed effects. This led to PA increases of 47.2% and 27.8%, respectively. When both markers were included together as fixed effects, the model’s PA still improved, but to a comparatively lower extent than the top SNP alone, achieving a 30.6% increase (Fig. 3). The de novo approach for fruit PM revealed a similar trend as for the historic SNPs, with up to a 35% increase in PA compared to the GBLUP model (Fig. 3 and Supplementary Table 1).

Across all models, the resistance-associated allele frequencies remained consistently balanced (ranging from 45.4% to 60.3%), suggesting that the observed gains in PA were supported by maintained genetic variance in the population and were not limited by allele fixation within the cross-validation folds (Supplementary Fig. 3).

### Family-wise prediction analysis

**Fig. 4:**
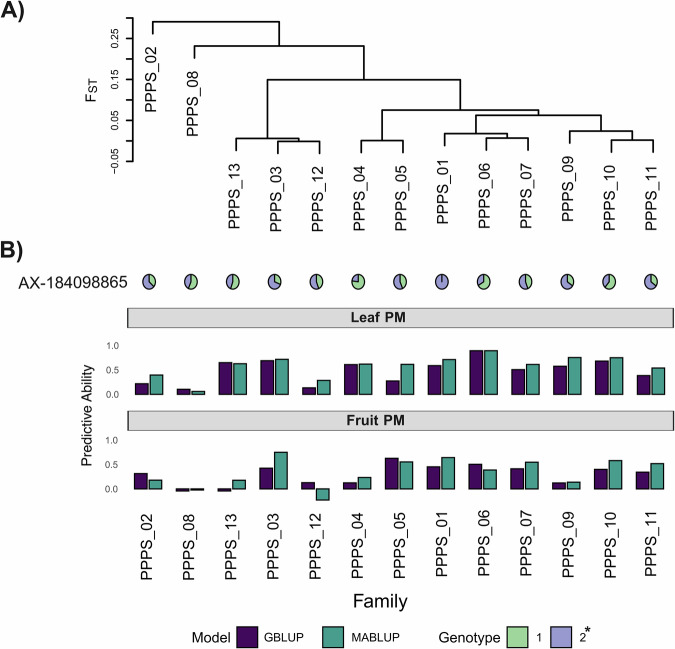
Family clustering of the ReC population by genetic distance and family-wise mean predictive abilities (PAs) of the Genomic Best Linear Unbiased Prediction (GBLUP) and the top-SNP-based Marker-assisted Genomic Best Linear Unbiased Prediction (MABLUP) models. Pairwise genetic distances were calculated as Fixation index (*F*_ST_) values, with scale shown on the left side of the dendrogram (**A**). Pie charts showing the allelic class distribution of the top historic SNP used as a fixed effect in the MABLUP model for both leaf and fruit PM resistance. Bar plots showing the PAs of GBLUP and MABLUP models for each progeny, the upper value being for leaf and the lower value for fruit powdery mildew (PM) resistance, and the asterisk (*) in the Genotype legend indicates the resistance-associated allele (**B**).

The structured ReC population was divided into four clusters in our previous population structure analyses (Rehman et al. 2024). The two largest clusters contain most of the families, and their progenies share parents with at least two of the other progenies of the same cluster, while between the clusters only the recurrent *F. virginiana* parents were shared (Table 1 and Supplementary Table 2). Two more distinct progenies, PPPS_02 and PPPS_08, each formed their own cluster (Table 1 and Supplementary Table 2). Pairwise *F*_ST_ analyses for progeny relatedness confirmed both this clustering, and the more detailed pedigree structure within the two largest clusters (Fig. 4A, Dataset, 10.5281/zenodo.20505163). The allele ratios of the historic top and focal SNPs (Fig. 4B and Supplementary Fig. 4, respectively) varied between the families.

**Table 1:** Predictive abilities (PAs) and narrow-sense heritability estimates (*h*^*2*^) of leaf and fruit PM resistance in a sib family prediction scenario using Genomic Best Linear Unbiased Prediction (GBLUP) model.

Training	Testing family	DAPC-based genetic cluster	*F. virginiana* recurrent parent	Full pedigree (Fc × Fv)	Testing family size	*h*^*2*^ leaf PM	*h*^*2*^ fruit PM	Leaf PM PA	Fruit PM PA
All other families	PPPS_11	2	RV-1	(SC × 2 MAR 1A) × (MR 10 × RH 23)	32	0.45	0.34	0.38	0.34
All other families	PPPS_10	2	RV-3	(SC × 2 MAR 1A) × (RH 30 × MR 10)	25	0.42	0.21	0.68	0.40
All other families	PPPS_09	2	RV-1	(SC × 2 BRA 1A) × (MR 10 × RH 23)	27	0.58	0.11	0.58	0.12
All other families	PPPS_07	3	RV-1	(NAH 3 × 2 MAR 1A) × (MR 10 × RH 23)	23	0.43	0.27	0.50	0.41
All other families	PPPS_06	3	RV-3	(NAH 3 × 2 MAR 1A) × (RH 30 × MR 10)	13	0.45	0.12	0.89	0.50
All other families	PPPS_01	3	RV-1	(HM1 × CFRA 24) × (MR 10 × RH 23)	22	0.49	0.22	0.59	0.45
All other families	PPPS_05	3	RV-1	(NAH 3 × CFRA 372) × (MR 10 × RH 23)	25	0.50	0.07	0.27	0.63
All other families	PPPS_04	3	RV-3	(NAH 3 × CFRA 372) × (RH 30 × MR 10)	26	0.63	0.18	0.60	0.12
All other families	PPPS_12	2	RV-1	(SC × RCP-37) × (MR 10 × RH 23)	13	0.41	0.00	0.13	0.13
All other families	PPPS_03	2	RV-1	(HM1 × RCP-37) × (MR 10 × RH 23)	9	0.38	0.52	0.69	0.43
All other families	PPPS_13	2	RV-3	(SC × RCP-37) × (RH 30 × MR 10)	23	0.51	0.26	0.65	-0.04
All other families	PPPS_08	1	parent of RV-3	CFRA 24 × RH 30	30	0.34	0.13	0.10	-0.04
All other families	PPPS_02	4	unrelated	complex hybrid × Frederick 9	30	0.48	0.20	0.22	0.31

Of the four genetic clusters, clusters 2 and 3 are the largest, sharing a recurrent *F. viriginiana* parent but having different sets of *F. chiloensis* parents.

Next, we tested if families that are not present in the training set could be predicted by models trained by related families and how reliable the predictions from the wider population were for the less connected families: the phenotypes for each of the 13 ReC families were predicted separately, while the remaining families served as the training set. The sib-prediction results showed that ReC families with higher genetic relatedness achieved better PAs than the random forward prediction (Table 1, Fig. 4B and Supplementary Table 3) regardless of whether significant historic SNPs were included as fixed effects. The combined-year *h*^2^ for Mean leaf PM resistance was estimated as 0.66, and for fruit PM resistance as 0.41. Family-specific *h*^2^ estimates ranged from 0.46 to 0.76 for leaf PM and from 0 to 0.68 for fruit PM resistance (Table 1).

Notably, families such as PPPS_03, PPPS_04, PPPS_06, PPPS_10, and PPPS_13 had PAs exceeding 0.6 for leaf PM resistance. For fruit PM resistance, families PPPS_01, PPPS_03, PPPS_05, PPPS_06, PPPS_07, and PPPS_10 had PAs above 0.4. In contrast, families with lower genetic relatedness—such as PPPS_02 and PPPS_08, which formed distinct clusters in the population structure analysis (Rehman et al. 2024), had lower PAs for both the traits, and for both GBLUP or MABLUP prediction approaches (Fig. 4 and Supplementary Fig. 4). Amongst the more closely related families, PA generally exceeded 0.5 for leaf and 0.3 for fruit PM resistance (Table 1), in agreement with genetic relatedness being a key factor influencing the performance of standard GBLUP model (Pereira et al. 2018).

### Cross-environment GP

**Fig. 5:**
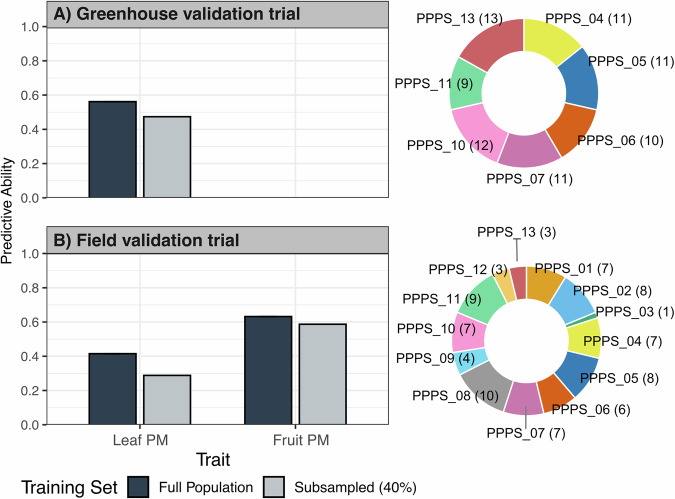
Cross-environment genomic prediction. Predictive abilities (PAs) of Genomic Best Linear Unbiased Prediction (GBLUP) models to predict new individuals in greenhouse validation trial (**A**) and field validation trial (**B**). Bar plots compare model PAs trained with the full ReC population (dark) versus a random 40% subset (light). The donut charts on the right illustrate the number of individuals from different ReC families present in the corresponding validation trial.

The analyses of phenotypic correlations between the initial ReC field trial and the two validation trials across different environments indicated moderate to strong consistency in disease severity: the Pearson’s correlations (*p* < 0.05) between the ReC experiment and the greenhouse (GH) validation trial were 0.58 for leaf PM, while correlations with the validation field trial were 0.75 for leaf PM and 0.65 for fruit PM. The subsets of plant material tested differed between the validation experiments: seven ReC families of the largest pedigree-based clusters were represented in the GH trial, while all of the ReC families were represented in the field validation trial (Fig. 5).

Our GBLUP model demonstrated varying levels of PAs when predicting new individuals across two environments under a strict forward-prediction scheme. When training the model with the full ReC population excluding any phenotypes from the respective test sets, the PAs were 0.56 for leaf PM in the GH validation trial, while 0.41 for leaf PM and 0.63 for fruit PM in the field validation trial (Fig. 5). Next, to mimic a realistic breeding scenario, the training set was further restricted. Based on the random sampling results described above, we applied randomly sampled 40% subset of the ReC population for training and ensured no overlap between individuals in the training set and those in the respective validation sets. As expected, this reduction in training set size led to a decrease in PAs; however, predictions remained moderately accurate for leaf PM in the GH and for fruit PM in the field validation trial (Fig. 5). Using the 40% subset of the 2020–2022 ReC individuals as training data and predicting all new individuals in the GH and field validation trials, the GBLUP model achieved mean PAs of 0.47 for leaf PM in the GH and 0.29 for leaf PM and 0.59 for fruit PM in the field validation trial.

## Discussion

The ReC strawberry population used in this study represents a pre-breeding resource specifically developed to reintroduce diversity from the garden strawberry’s wild progenitor species, *F. virginiana* and *F. chiloensis*. In our previous work using this pre-breeding population, multiple QTLs were identified for key horticultural and adaptive traits (Rehman et al. 2024), as well as a major QTL for leaf and fruit PM (Rehman et al. 2025), highlighting its potential as a source of novel alleles for breeding. In this context, the present study explores how GP can be directly integrated into a strawberry pre-breeding pipeline to accelerate the identification and utilization of superior individuals. GP method reduces the need for extensive multi-year phenotyping and thereby allows breeders to prioritize promising individuals for genetic advancement by enabling early selections, eventually leading to strawberry cultivars with durable PM resistance (Cockerton et al. 2018). The incorporation of trait-associated loci into prediction models further enhances the model’s PA, particularly for traits influenced by major QTLs (Bernardo 2014). From a breeding perspective, this approach facilitates the transfer of favorable alleles from the pre-breeding populations, i.e., the ReC population, into elite strawberry germplasm, thereby supporting the development of cultivars with improved disease resistance and adaptability. Ultimately, this study highlights the potential for genomic-informed strategies to streamline the integration of wild genetic resources into modern breeding pipelines, offering a methodology that is also adaptable to other complex traits and in other crop species.

PM resistance in strawberry is a complex trait controlled by multiple QTLs with varying effects. In this study, we carried out GP for PM resistance traits in a pre-breeding population where both major and minor resistance QTLs are present. While GWAS identifies individual marker–trait associations, GP complements this by estimating breeding values using genome-wide marker information, thereby accounting for the combined effects of all contributing loci. We demonstrated that integrating both GWAS and GP enables the development of marker-assisted prediction models that can be directly applied in pre-breeding to accelerate genetic gain. Consequently, it provides a practical pathway for efficient introgression of PM resistance from wild-derived materials into the cultivated gene pool of strawberry. In this study, GEBVs for different traits predicted by the GBLUP model achieved PAs ranging from 0.36 to 0.56 for fruit and leaf PM resistances, respectively. These results align with previous studies, which report similar PAs, ranging from 0.25 to 0.60 for leaf PM and up to 0.37 for fruit PM (Tapia et al. 2022; Lynn et al. 2024). Furthermore, different strategies to incorporate GWAS loci into prediction models were explored, which consistently resulted in improved PAs compared to standard GBLUP.

### Factors affecting predictive ability

Several factors influence the GP model’s PA, including the size and structure of the training population, marker density, linkage disequilibrium (LD), trait heritability, and the precision of phenotypic measurements (Nakaya and Isobe 2012; Alemu et al. 2024). Also, our study showed that training set size and marker set density affected the PA of GP models for PM resistance in both leaves and fruits of the strawberry. The consistent PA plateau observed for both traits beyond 40% of the population size (*n* ≈ 120 individuals) suggests that this threshold represents the point where most of the effective loci are adequately sampled in this wild-derived genetic background. Similarly, the trends observed in marker set size effects further highlight the distinct genetic control of these resistance traits. For leaf PM resistance, the PA improved progressively with increasing marker density up to approximately 40% of the SNPs (*n* ≈ 4156), beyond which no substantial gains were observed. This pattern indicates that leaf PM resistance can be effectively captured with a moderate-density SNP marker panel. Recently, medium-density genotyping platforms (3 K and 5 K) in strawberry were made available for low-cost genomic predictions (Hardigan et al. 2023). A subset of the 50 K markers used in our study can be incorporated with such platforms to generate new marker panels in the future for genomic selection for PM resistance in garden strawberry.

The noted absence of any meaningful relationship between marker number and the model’s PA for fruit PM resistance suggests distinct genetic architectures of these two traits regardless of our earlier report about a common major QTL for both leaf and fruit PM (Rehman et al. 2025). While the major QTL common to fruit and leaf PM resistances (PVE > 40% for both traits, Rehman et al. 2025) may contribute baseline resistance in both tissues, its predictive value appears modulated by tissue-specific minor QTLs. Previous research has also identified patterns of developmentally regulated resistances in the fruit and leaf organs of strawberry (Asalf et al. 2014). This interpretation aligns with Lynn et al. (2024), who observed lower PA for fruit PM and hypothesized tissue-specific resistance mechanisms. Together, these results suggest that the standard GBLUP model may have failed to capture the minor background QTLs for fruit PM. Such minor resistance QTLs are influenced by the environment, are less stable, and may express only in particular developmental stages (Young 1996), all of which need further investigation. It is also important to note that, unlike the leaf PM phenotypic data, which were based on time-series measurements and collected over 3 years, the fruit PM data were recorded as 1–2 observations per plant at the time of secondary berry ripening and for only 2 years. These differences in phenotyping protocols between the two traits may partly explain the observed patterns of low heritability estimates and PAs of the tested models in this study.

Our findings about the thresholds for training population size and marker density have practical importance for breeding programs, as they indicate that carefully constructed training sets of moderate size and medium to low density marker sets can capture sufficient genetic variation and can potentially reduce the cost for operational genomic selection. However, the contrasting patterns observed between leaf and fruit PM also highlight that different traits may require different GP strategies, including model choices and the potential inclusion of non-additive or interaction terms. Therefore, our findings also suggest that the most efficient strategy may require separate optimization for foliar versus fruit resistance in strawberry, even when targeting the same pathogen for resistance breeding.

### Utilizing GWAS findings enhanced the PA of GP models

The traditional GBLUP model assumes that all markers contribute equally to trait variance, applying uniform shrinkage toward zero. While this assumption effectively captures the polygenic nature of traits, it may underestimate the effects of major QTLs (De Los Campos et al. 2013). To address this limitation, it has been recommended to include information in the model when major genes controlling the trait are known (Bernardo 2014). Therefore, we employed a marker-assisted GBLUP model by incorporating significant historic SNPs from the major QTL region identified through our previously reported GWAS of PM resistance in the same ReC population (Rehman et al. 2025) as fixed effects. This integration substantially improved the PA of GBLUP for both leaf and fruit PM resistance, demonstrating the utility of MABLUP models. However, because the historic SNP GWAS relied on the full ReC dataset, the resulting fixed-effect SNPs were not independent of the individuals being predicted, which likely inflated PA estimates due to overfitting (McGowan et al. 2021; Alemu et al. 2023). To address this issue, we implemented a 5-fold cross-validation scheme in which GWAS was repeated exclusively within each training fold, ensuring that de novo SNP discovery remained fully independent of the corresponding test fold. When these fold-specific de novo SNPs were used as fixed effects in the MABLUP models, the resulting MABLUP PAs were more robust, providing unbiased evidence that incorporating trait-associated loci genuinely improves GP performance.

Notably, our de novo GWAS repeatedly identified the same top SNP (AX.184098865) that was previously detected in the historic GWAS (Rehman et al. 2025), supporting the stability of the association signal. When alternative SNPs were selected across folds, they consistently mapped to the same major QTL region on chromosome 3B, indicating that the underlying QTL was reliably captured regardless of the training subset used for SNP discovery. While the assumption of equal marker variance in GBLUP may hold in other cases, such as traits that are more closely following an infinitesimal model (Fisher 1918; Barton et al. 2017), our findings showed that accounting for the known genetic architecture of PM resistance led to better performance across all tested scenarios by assigning greater weight to major loci and thus better reflecting the reality. This aligns well with the case of disease resistance traits that are often influenced by both large-effect QTLs and a complex background of small-effect QTLs (Poland and Rutkoski 2016).

Numerous previously published simulation and empirical studies have been conducted in other crops, in which significant markers were integrated as fixed effects and enhanced PA. These include: *Septoria tritici* blotch and Fusarium head blight resistance; yield and yield stability in wheat (Arruda et al. 2016; Sehgal et al. 2020; Alemu et al. 2021; Zakieh et al. 2023; Nannuru et al. 2024); growth traits in eucalyptus (Tan and Ingvarsson 2022); simulated complex traits in maize and sorghum (Rice and Lipka 2019); rust resistance in sugarcane (Islam et al. 2021). However, it is important to consider that many of these studies used non-independent GWAS results derived from the full dataset, including the test individuals, which may have led to inflated PAs in those models. In contrast, other studies adopted more reliable strategies, similar to our de novo approach, and reported PAs more comparable to those observed under independent validation scenarios (Spindel et al. 2016; Alemu et al. 2023; Munyengwa et al. 2025; Yu et al. 2026).

Nevertheless, our findings of increased PA by marker-assisted GBPLUP contrast with a recent study in strawberry, which reported no such improvements when applying similar approaches to *Neopestalotiopsis* resistance prediction (Alam et al. 2024). This suggests pathogen-specific genetic resistance architectures or the strength of LD may influence the effectiveness of such marker-assisted GP models in different populations.

Furthermore, we found that adding multiple historic SNPs from QTLs beyond our top GWAS hit did not improve predictions (results not shown), consistent with observations in maize/sorghum where excessive fixed effects sometimes decreased model PA (Rice and Lipka 2019). This likely occurs because: (1) secondary SNPs may have smaller effects that get obscured by statistical noise, or (2) LD between top SNPs makes additional markers redundant in the model, causing over-parameterization. Therefore, we suggest finding a “sweet spot” in GWAS-assisted prediction models—including just one to two major-effect SNPs could maximize PA without running into over-parameterization.

### Family-wise forward predictions and potential sources of resistance

Overall, family-wise forward predictions showed that multiple ReC families achieved PAs above 0.55 and 0.40 for the prediction of leaf and fruit PM resistance, respectively, when all other families were used for model training. However, interesting patterns were observed while comparing PAs of the same families for both traits simultaneously. This contrast was most pronounced in PPPS_05, which showed poor PA for leaf PM resistance (0.27), but one of the highest for fruit PM resistance (0.63). On the other hand, PPPS_04 showed an opposite trend, with higher PA for leaf PM resistance (0.60) and lower for fruit PM resistance (0.12). The observed difference between the PAs of leaf and fruit PM resistance further supported the idea that the genetic architectures of these traits might be different. This is consistent with the identification of a fruit-specific QTL on chromosome 4B (Rehman et al. 2025), and with prior suggestions of tissue-specific regulation of PM resistance responses in strawberry (Asalf et al. 2014; Lynn et al. 2024).

The GP performance across different families in this study can be further interpreted through the lens of the ReC population’s unique pedigree, which combines historically characterized wild accessions of *F. virginiana* and *F. chiloensis*. Previous trials established that all four *F. virginiana* grandparents—MR10, RH23, RH30, and Frederick 9—showed strong PM resistance in field trials performed in Florida (Kennedy et al. 2013). However, the progenies of MR10 when crossed with *F. × ananassa* cultivars showed susceptibility, and RH23 progenies showed moderate resistance, while Frederick 9 produced highly resistant offspring in field trials conducted in Minnesota (Hancock et al. 2002).

Notably, the genetic distance between training and testing clusters based on F_ST_ values had previously been shown to have a significant effect on the PA of quantitative traits (Scutari et al. 2016). They found that the model PA decays in a linear fashion when the distance between the training and testing sets increases. We observed a similar pattern in our ReC population, where both a single descendant family of Fredrick 9 (PPPS_02) and another family (PPPS_08) derived from a *F. chiloensis* landrace (CFRA 24) had among the lowest PAs, due to almost unshared parentage with any other families of the ReC population that were used as the training set. On the other hand, families that had shared parental origin and made the largest F_ST_-based cluster had considerably high PAs, with the exception of family PPPS_12. In our dataset, this exception can be explained by the low number of individuals (*n* = 13) in this family per se, rather than any lack of shared parents (Osorio et al. 2021). Moreover, the GBLUP model has been shown to be influenced by the relationship among individuals and has a limited ability to effectively utilize LD information (Habier et al. 2007). Therefore, the lack of PA may also imply a poor statistical architecture of our GP model when applied to genetically distant, or particular, families without providing any explicit additional information, such as pedigree or population structure covariates (Zhou et al. 2016; Rio et al. 2021).

Importantly, the ReC population extends beyond its role as an experimental population, its structured pedigree providing a practical template that breeding programs can adapt for the introgression and evaluation of powdery mildew resistance, among other traits of interest in garden strawberry. Because the grandparental accessions of the ReC population are publicly available from gene banks via their PI identifiers listed in Rehman et al. (2024), breeders may have access to the same germplasm and reconstruct similar families with favorable traits. When combined with the known QTLs reported in Rehman et al. (2024, 2025) and the various prediction models documented in this study, species reconstruction from progenitor species is a great approach for strawberry improvement. Together, these datasets offer a framework for selecting parents, assembling training sets, and devising genome-informed breeding strategies for strawberry breeders globally. Furthermore, our GP results support the use of comparable strategies for traits in other crops that combine major-effect QTLs with a wider polygenic landscape.

### Cross-environment predictions

Our results demonstrate that GBLUP can generate robust predictions for PM resistance in new individuals across diverse environments in both leaf and fruit tissues. By intentionally excluding the validation individuals from the training set, we simulated a realistic breeding scenario where only genomic information is used to predict the performance of new individuals in two new independent environments. The highest PA for leaf PM in the greenhouse validation trial (0.56) was achieved despite representation from only seven of the thirteen ReC families. This suggests that the exclusion of the most genetically distant families (PPPS_02 and PPPS_08) from the validation dataset resulted in improved prediction for the remaining genotypes—as also suggested by sib family prediction results, where the leaf PM model performed better for the families of the largest ReC cluster. These findings are consistent with prior studies in strawberry and other crops, where stable genomic predictions have been observed across years and populations (Alemu et al. 2021; Montesinos-López et al. 2024), although PAs often decline when individuals shared between training and validation sets are excluded (Osorio et al. 2021).

However, the PAs for leaf PM in the field validation trial were consistently lower than in the greenhouse trial across both training sizes. This pattern likely reflects greater environmental heterogeneity in the field and across years, which introduces additional sources of phenotypic variation not captured by the GBLUP model. Conversely, the higher PAs for fruit PM in the field compared to leaf PM suggest that fruit-specific resistance may be more stable across the ReC and field validation environments.

The decline in PA for the 40% ReC subset relative to the full ReC training set reflects the expected reduction in the information given to the model, but still demonstrates that GP is feasible under such constraints and in a more realistic breeding scenario. In both cases, the cross-environment PA remained substantial, indicating that the genomic relationship matrix successfully captured sufficient covariance to bridge the training and the testing individuals. Despite differences in the representation of different ReC families across the two validation trials, GBLUP maintained strong predictive performance, highlighting the robustness of the models and suggesting their utility for forecasting trait performance across diverse environments. Our results further indicate that standard GBLUP, even without explicit modeling of genotype-by-environment interactions, can capture stable signals of PM resistance across environments. At the same time, we acknowledge that genotype-specific responses to environmental variation, soil type, or microclimatic differences—factors not captured by a simple additive genomic model—could potentially improve model performance if explicitly incorporated in future modeling frameworks.

## Conclusions

The present study evaluated predictive abilities across a structured multi-family strawberry pre-breeding population using both unassisted and marker-assisted genomic prediction models for PM resistance in both leaf and fruit tissues and yielded, overall, encouraging results within and across different environments. We found that even modest numbers of markers and relatively small training sets were sufficient to achieve considerable predictive abilities for both traits, enabling improved selection of resistant wild-derived materials at potentially reduced cost. The tested models performed better when significant SNPs were explicitly included as fixed effects. Furthermore, we demonstrated that genetic relatedness among families enhanced predictive performance and could guide the design of training sets optimized for PM resistance traits in strawberry. Consequently, we recommend that effective pre-breeding programs could actively structure training sets to include highly representative pedigrees, rather than relying on random sampling of wild accessions, to prevent severe drop-offs in model performance. Future efforts could benefit from models that explicitly account for population structure and environmental covariates, with the aim of improving predictive abilities in structured multi-family populations.

## Supplementary information

Supplmentary File

## Data Availability

The dataset containing the genotypic data, results from various predictions, and the fixation index analyses are available at 10.5281/zenodo.20505163.
