# Supplementary material for: Genomic prediction of wild-derived powdery mildew resistance for strawberry (Fragaria × ananassa) pre-breeding
Source: Heredity (Edinb). 2026 Jun 17;135(7):525–36. doi: 10.1038/s41437-026-00857-2 (PMC13451407; doi:10.1038/s41437-026-00857-2)
Supplement: Supplementary file 1 [file 41437_2026_857_MOESM1_ESM.docx]

**Supplementary Figures and Tables**

**Supplementary Figure 1.** Stacked Manhattan plots for GWAS conducted on five training subsets of the ReC population for leaf PM. The red horizontal lines indicate the genome-wide significance level (−log10(p) = 5.61) and significant SNPs are indicated as red dots. SNP effect boxplots for the top two significant SNPs discovered in each fold are plotted on the right.

**Supplementary Figure 2.** Stacked Manhattan plots for GWAS conducted on five training subsets of the ReC population for fruit PM. The red horizontal lines indicate the genome-wide significance level (−log10(p) = 5.61) and significant SNPs are indicated as red dots. SNP effect boxplots for the top two significant SNPs discovered in each fold are plotted on the right.

**Supplementary Figure 3.** Pie charts illustrating the allele frequency distributions of significant SNPs for leaf PM (above) and fruit PM (below). The left side indicates historic SNPs from previously published QTLs (Rehman et al. 2025) and the right side indicates *de novo* SNPs, with an asterisk (*) indicating the resistance-associated allele.

**Supplementary Table 1.** Mean predictive abilities (PA) with standard errors (SE) for leaf and fruit PM resistance for GBLUP and MABLUP model with *de novo* SNP markers as fixed effect. For MABLUP, the percentage increase in PA compared to respective GBLUP is also mentioned.

| **Trait** | **Model** | **Approach** | **Fixed_SNPs** | **PA** | **SE** | **% increase in PA** |
| --- | --- | --- | --- | --- | --- | --- |
| **Leaf PM** | GBLUP | - | - | 0.57 | 0.03 | - |
|  | MABLUP | *de novo* | SNP1 | 0.66 | 0.02 | 13.9 |
|  |  | *de novo* | SNP2 | 0.63 | 0.01 | 9.7 |
|  |  | *de novo* | SNP1 + SNP2 | 0.65 | 0.02 | 13.6 |
| **Fruit PM** | GBLUP | - | - | 0.33 | 0.03 | - |
|  | MABLUP | *de novo* | SNP1 | 0.42 | 0.06 | 25 |
|  |  | *de novo* | SNP2 | 0.45 | 0.06 | 35.4 |
|  |  | *de novo* | SNP1 + SNP2 | 0.44 | 0.07 | 33.5 |

**Supplementary Table 2.** Crossing schema used to create the ReC population.

|  |  | | ***F. virginiana* parent** | | | |
| --- | --- | --- | --- | --- | --- | --- |
|  |  | **RV-1** | | **RV-3** | **RH30**  (a parent of RV-3) | **Frederick 9** |
| ***F. chiloensis* parent** | **RC-5** | **PPPS_09** | |  |  |  |
|  | **RC-1** | **PPPS_11** | | **PPPS_10** |  |  |
|  | **RC-6** | **PPPS_07** | | **PPPS_06** |  |  |
|  | **RC-a** | **PPPS_01** | |  |  |  |
|  | **RC-c** | **PPPS_05** | | **PPPS_04** |  |  |
|  | **RC-11** | **PPPS_12** | | **PPPS_13** |  |  |
|  | **RC-b** | **PPPS_03** | |  |  |  |
|  | **CFRA 24**  (a parent of RC-a) |  | |  | **PPPS_08** |  |
|  | **RC-8** |  | |  |  | **PPPS_02** |

**Supplementary Table 3.** Predictive ability of leaf and fruit PM resistance in a sib family prediction scenario with MABLUP model, including different Historic SNPs as fixed effects.

| **MABLUP** | | **Leaf PM** | | | **Fruit PM** | | |
| --- | --- | --- | --- | --- | --- | --- | --- |
| **Training** | **Testing family** | **AX.184098865** | **AX.184895875** | **AX.184098865**  **+**  **AX.184895875** | **AX.184098865** | **AX.184327796** | **AX.184098865**  **+**  **AX.184327796** |
| All other families | PPPS_11 | 0.54 | 0.27 | 0.49 | 0.53 | 0.52 | 0.52 |
| All other families | PPPS_10 | 0.75 | 0.55 | 0.72 | 0.58 | 0.58 | 0.58 |
| All other families | PPPS_09 | 0.75 | 0.71 | 0.77 | 0.22 | 0.14 | 0.17 |
| All other families | PPPS_07 | 0.61 | 0.58 | 0.61 | 0.54 | 0.55 | 0.55 |
| All other families | PPPS_06 | 0.89 | 0.90 | 0.89 | 0.38 | 0.39 | 0.37 |
| All other families | PPPS_01 | 0.71 | 0.69 | 0.71 | 0.58 | 0.64 | 0.61 |
| All other families | PPPS_05 | 0.61 | 0.46 | 0.62 | 0.55 | 0.55 | 0.53 |
| All other families | PPPS_04 | 0.62 | 0.55 | 0.62 | 0.33 | 0.23 | 0.30 |
| All other families | PPPS_12 | 0.29 | 0.33 | 0.31 | -0.27 | -0.23 | -0.30 |
| All other families | PPPS_03 | 0.72 | 0.75 | 0.71 | 0.75 | 0.75 | 0.76 |
| All other families | PPPS_13 | 0.63 | 0.61 | 0.61 | 0.18 | 0.18 | 0.19 |
| All other families | PPPS_08 | 0.06 | 0.09 | 0.09 | 0.04 | -0.02 | 0.03 |
| All other families | PPPS_02 | 0.40 | 0.12 | 0.40 | 0.30 | 0.18 | 0.33 |

**Supplementary Figure 4.** Family clustering of the ReC population by genetic distance and mean predictive abilities (PAs) per family. Pairwise genetic distances were calculated as F_ST_ values, with scale shown on the left side of the dendrogram (**A**). Pie charts showing the allelic class distribution of the focal SNPs used as a fixed effect in the MABLUP model for both leaf and fruit PM resistance. Bar plots showing the PAs of GBLUP and MABLUP models for each progeny, the upper value being for leaf and the lower value for fruit PM resistance, and the asterisk (*) dagger (†) in the Genotype legend shows resistance-associated allelic combinations for leaf and fruit PM focal SNPs, respectively (**B**).
